# Interferometric microscopy study of the surface roughness of Portland cement under the action of different irrigants

**DOI:** 10.4317/medoral.19082

**Published:** 2013-05-31

**Authors:** Maria L. Ballester-Palacios, Esther M. Berástegui-Jimeno, Neus Parellada-Esquius, Carlos Canalda-Sahli

**Affiliations:** 1 Associate Professor of Dental Pathology and Therapeutics. Investigator of the IDIBELL Institute. School of Dentistry. University of Barcelona. Barcelona. Spain; 2 Professor of Dental Pathology and Therapeutics. Investigator of the IDIBELL Institute. School of Dentistry. University of Barcelona. Barcelona. Spain; 3 Physician specialized in Epidemiology and Public Health. Catalan Health Institute. Barcelona. Spain; 4 Chairman of Dental Pathology and Therapeutics. Investigator of the IDIBELL Institute. School of Dentistry. University of Barcelona. Barcelona. Spain

## Abstract

Objectives: Some investigations suggested common Portland cement (PC) as a substitute material for MTA for endodontic use; both MTA and PC have a similar composition. The aim of this study was to determine the surface roughness of common PC before and after the exposition to different endodontic irrigating solutions: 10% and 20% citric acid, 17% ethylenediaminetetraacetic (EDTA) and 5% sodium hypochlorite. Study Design: Fifty PC samples in the form of cubes were prepared. PC was mixed with distilled water (powder/liquid ratio 3:1 by weight). The samples were immersed for one minute in 10% and 20% citric acid, 17% EDTA and 5% sodium hypochlorite. After gold coating, PC samples were examined using the New View 100 Zygo interferometric microscope. It was used to examine and register the surface roughness and the profile of two different areas of each sample. Analysis of variance (ANOVA) was carried out, and as the requirements were not met, use was made of the Kruskal-Wallis test for analysis of the results obtained, followed by contrasts using Tukey’s contrast tests. Results: Sodium hypochlorite at a concentration of 5% significantly reduced the surface roughness of PC, while 20% citric acid significantly increased surface roughness. The other evaluated citric acid concentration (10%) slightly increased the surface roughness of PC, though statistical significance was not reached. EDTA at a concentration of 17% failed to modify PC surface roughness. Irrigation with 5% sodium hypochlorite and 20% citric acid lowered and raised the roughness values, respectively. Conclusions: The surface texture of PC is modified as the result of treatment with different irrigating solutions commonly used in endodontics, depending on their chemical composition and concentration.

** Key words:**MTA, Portland cement, citric acid, ethylenediaminetetraacetic acid, sodium hypochlorite, surface roughness.

## Introduction

Several studies have compared mineral trioxide aggregate (MTA) with Portland cement and the findings suggest that both show almost identical macroscopically, microscopically, and by X-ray diffraction analysis (1).

Other study affirms that Portland cements contain the same chemical elements as MTA (2,3). This suggests that Portland cement has the potential to be used as a less expensive root-end-filling material in dental practice (4).

Camilleri et al. (5) determined the composition of the two commercially available MTA formulations (ProRoot MTA, Dentsply Tulsa, Tulsa, OK, USA), gray and white. Dispersion analysis showed white MTA (WMTA) to be composed mainly of calcium, silica, bismuth and oxygen, while gray MTA (GMTA) also contained iron and aluminum. X-ray diffraction analysis demonstrated that MTA was composed of tricalcium silicate and bismuth oxide, while GMTA was found to be composed of tricalcium silicate, dicalcium silicate and bismuth oxide. Thus, the commercial versions of MTA were shown to have a composition similar to that of Portland cement, widely used as a binder for materials employed in the building industry, with the exception that MTA contained bismuth oxide to enhance its radiopacity (6). In ProRoot MTA, the amount of gypsum is approximately half of that of the Portland cements. ProRoot MTA consists of fewer toxic heavy metals like copper (Cu), manganese (Mn) and strontium (Sr), which might reduce rejection, inflammation or other allergic reactions when applied to the patient. The Portland cements are composed of particles with a wide range of size, whereas ProRoot MTA particles are smaller and uniform in size (7).

Irrigating solutions used in endodontics, such as ethylenediaminetetraacetic acid (EDTA) and citric acid, designed to eliminate the smear layer, are sufficiently aggressive to demineralize intraradicular dentin (8-10). These irrigants generate a sufficiently acid environment (pH=2) to cause serious deterioration of Portland cement (11). Thus, when citric acid or EDTA are used during second treatment or retreatment of root canals presenting perforations repaired with MTA, they could affect the surface of this material altering its properties and its roughness. However, few studies have analysed the effect of these irrigants upon the surface of MTA in terms of cement corrosion and dissolution.

During endodontic therapy various irrigating solutions at different concentrations and duration are used. These chemical solutions may affect the setting reaction of MTA.

After a final flushing with a chemical irrigant, some amount of the irrigating solution may remain in the root canal space, which may affect the properties of MTA.

The present study investigates and compares the surface roughness of Portland cement (PC) before and after treatment with different irrigating solutions commonly used in endodontic.

## Material and Methods

Fifty cubic samples (1cm x 1cm x1cm) of common Portland Cement (PC) (Cemex, Grey cement. Sant Feliu del Llobregat. Barcelona. Spain) were prepared as follows.

Cubic silicone molds (Empress Penta 2 H Quick, 3M ESPE AG, Seefeld, Germany) were made setting the silicone while resin prisms of rectangular base (1cm x 1 cm) were placed at a depth of 1 cm. After prisms were removed, leaving spaces served as molds for the PC (Cemex, Grey Cement. Sant Feliu del Llobregat. Barcelona. Spain). A metal spatula was used for mixing PC in 3/1 proportion (cement/ water) on a glass plate to form a paste. The cement was added and carefully compacted within the silicone molds using the spatula and condensers. The samples were allowed to dry for 48 hours at room temperature. The preparations were removed from the molds and numbered from 1 to 50. Then, the samples were immersed in glass recipients containing the different irrigating solutions. The following irrigants were used: 5% sodium hypochlorite, 10% and 20 % citric acid and 17% EDTA, prepared in pharmacy according to magistral formula. All samples were exposed for one minute.

Five experimental groups were established. Group 1: samples 1-10, control, not exposed to the irrigating solutions. Group 2: samples 11-20, exposed to 5% sodium hypochlorite; Group 3: samples 21-30, exposed to 20% citric acid, Group 4: samples 31-40, exposed to 10% ci-tric acid, and Group 5; samples 41-50, exposed to 17% EDTA solution.

Both the treated and the untreated samples were posteriorly washed with distilled water for 5 minutes, shaking them gently to remove the traces of irrigating solution. The samples were left to dry for 48 hours and, then, were examined using the New View 100 interferometric microscope (Zygo, Middelefield, Connecticut, USA).

This microscope allows an accurately and reliably measure surface topography. Optical profilometry is a nondestructive, and noncontact measure surface metrology technique.

PC is a nonreflecting material; as a result, prior gold coating of the samples was carried out using the sputtering technique. Interferometric microscopy was used to examine and register the surface roughness and the profile of two different areas of each sample. The study parameters were PV (maximum roughness depth), Ra (mean roughness value) and Rms (roughness mean square value). Based on the data obtained, calculations were made of the variation in surface roughness of the surfaces treated with the different irrigating solutions versus those not exposed to the irrigants.

-Statistical analysis of the results. Analysis of variance, (ANOVA) was carried out, but as the requirements were not met, use was made of the Kruskal-Wallis test for analysis of the results obtained. The presence of significant differences between groups was evaluated using Tukey’s contrast tests (p ≤ 0.05).

## Results

 Table 1 shows the mean values of each parameter, together with the standard deviation, for the global samples analyzed and for each treatment group.

**Table 1:**
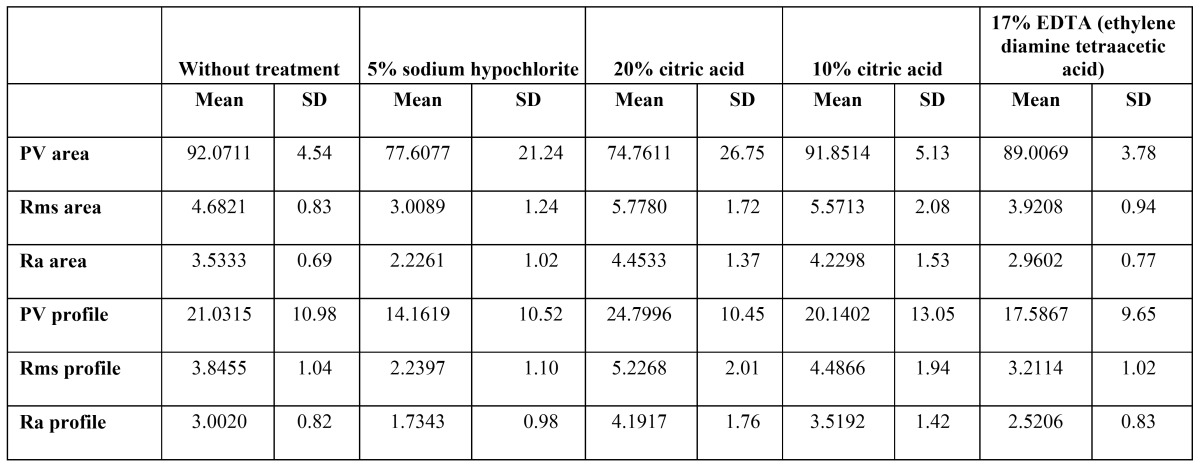
Mean and standard deviation(SD) values of each of the study measures, for the global samples analyzed and for each treatment group. PV (maximum roughness depth), Ra (mean roughness value) and Rms (roughness mean square value).

The PV area values for the samples exposed to 5% sodium hypochlorite were smaller than in the case of the untreated samples (p=0.035), according to Tukey’s contrast tests. Treatment with 20% citric acid in turn yielded significantly greater mean values versus the untreated samples (p=0.006).

The Rms area values differed significantly according to the type of treatment used (p<0.0001). Based on Tukey’s contrast tests, treatment with 5% sodium hypochlorite yielded lower values than in the case of the untreated samples (p=0.004).

The Ra area values likewise differed significantly according to the type of treatment used (p<0.0001). In the absence of treatment, the mean value was found to be greater than in the 5% sodium hypochlorite group (p=0.004).

The PV profile measurements differed significantly according to the type of treatment used (p=0.04). Tukey’s contrast tests revealed no differences in the measurements obtained after exposure to 20% citric acid and 5% sodium hypochlorite.

The Rms profiles measurements differed significantly according to the type of treatment used (p<0.0001). The values obtained after treatment with 5% sodium hypochlorite were lower than those recorded in the untreated group of samples (p=0.008). Differences were also seen with respect to the results obtained after treatment with 20% citric acid (p=0.034).

The Ra profiles measurements likewise differed significantly according to the type of treatment used (p<0.0001). Treatment with 5% sodium hypochlorite yielded lower values than in the case of the untreated samples (p=0.012). Differences were also seen with respect to the results obtained after treatment with 20% citric acid (p=0.022) ( Table 2).

**Table 2:**
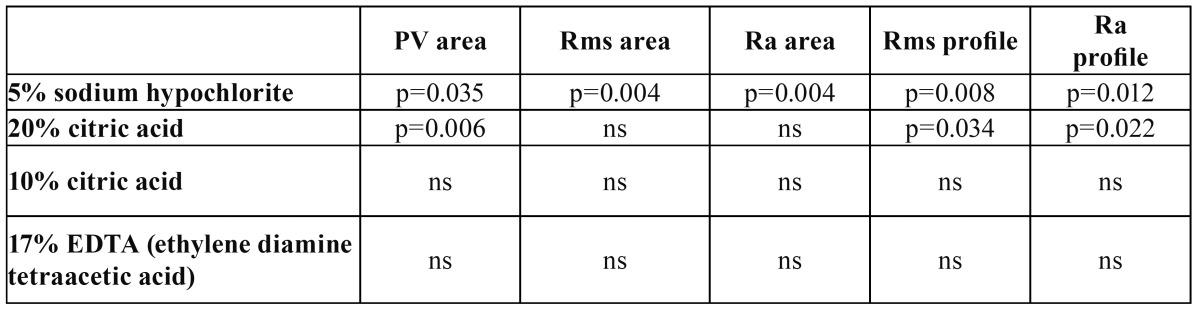
Significance level (p value) in the comparation of PV area, Rms area, Ra area. Rms profile and Ra profile between the samples with irrigant and the untreated samples (Tukey’s contrast). PV (maximum roughness depth), Ra (mean roughness value) and Rms (roughness mean square value).

## Discussion

Several authors have studied modifications of MTA by irrigating solutions commonly used in endodontics.

Yan et al. (12) evaluated the effects of 5.25% sodium hypochlorite, 2% chlorhexidine and EDTA–carbamide peroxide (Glide Prep) (Dentsply Maillefer, Ballaigues, Switzerland) upon MTA adhesion to dentin. No differences were observed between sodium hypochlorite and chlorhexidine, though in the case of Glide Prep adhesion was significantly affected. Glide File Prep is a combination of 15 %EDTA and 10%Carbamide in a water-soluble base. The acidic environment had an adverse effect on MTA-dentin bond strengths.

The influence of different irrigation protocols upon filtration after furcal repair with MTA and zinc oxide eugenol cement modified with ethoxybenzoic acid (Super-EBA). The study groups comprised 5.25% sodium hypochlorite, 5.25% sodium hypochlorite with EDTA, 5.25% sodium hypochlorite with doxycyclinecitric acid-detergent mixture (Biopure MTAD), and a non-irrigated group. EDTA and Biopure MTAD were seen to reduce the sealing effect of MTA and Super EBA(13,14).

Smith et al. (11) also examined the surface characteristics of white MTA in response to exposure to different irrigating solutions used: 1.3% sodium hypochlorite, 17% EDTA and Biopure MTAD (Dentsply Tulsa) in solution and evaluated calcium depletion. The exposure times were 1,3 and 5 minutes. The material exerting the greatest effect upon the MTA surface was found to be Biopure MTAD. The effect of Biopure MTAD ( pH=2) on the surface corrosion and dissolution of MTA would be related to our results with 20% citric acid.

One of the effects of irrigants in dentin is that it removes the smear layer(14).

Yildirim et al. (15) conducted an in vitro study of the effect of the smear layer upon apical filtration in teeth filled with white MTA. The authors concluded that apical filtration with MTA is less pronounced when the smear layer is present than when it is absent.

These results are in agreement with investigations which found that removal of smear layer by EDTA promotes an increase in dentinal permeability, which has a positive influence on microbial leakage (16).

Variations in the pH value, particularly because of bacterial-induced local metabolic acidosis or tissue inflammation, could possibly influence MTA physical and chemical properties (17).

It has been evaluated MTA microfiltration, using this material for apical filling exposed to a variety of acidic media during hydration. Significantly longer time was needed for filtration to occur in the samples stored at high pH values (18).

In another study, Saghiri et al. (19) evaluated the microstructure and surface hardness of WMTA (WMTA. Dentsply Tulsa) after exposure to a range of alkaline environments during hydration. The authors concluded that surface hardness and morphology can be influenced by different alkaline pH values.

Also the bond strength and the sealing ability of WMTA might be affected by alkaline pH (20,21).This should be taken into account particularly when alkaline residues remain within the canal.

Namazikhah et al. (22) studied the effect of pH upon the surface hardness (as determined using the Vickers test) and the microstructure of MTA (using scanning electron microscopy).Final maximum hardness corresponded to the surface exposed to pH 7.4, and minimum hardness to pH 4.4 with statistically significant differences between the two groups. It showed the porosity tended to increase with more acidic solutions.

Regarding the roughness-diminishing action of 5% sodium hypochlorite, a possible explanation would be dissolution of the outermost surface layer in a way similar to the situation observed by Bodanezi et al. (23) on fully immersing MTA and Portland cement rings in an aqueous medium.

The similarity of MTA and Portland cement, these effects might happen to MTA as well.

Based on the methodology used and the results obtained in this study, it can be concluded that 5% sodium hypochlorite applied for one minute through immersion significantly reduces the surface roughness of PC. In contrast, 20% citric acid applied in the same way significantly increases roughness, while 10% citric acid solution does so only slightly without reaching statistical significance. EDTA at a concentration of 17% does not modify the surface roughness of Portland cement. Thus, the PC surface is modified in different ways when using irrigating solutions commonly used in endodontics, depending on their chemical composition and concentration. Further studies are needed to better evaluate the importance of the action of these irrigating solutions in the clinical setting.

## References

[1] Islam I, Chng HK, Yap AU (2006). X-ray diffraction analysis of mineral trioxide aggregate and Portland cement. Int Endod J.

[2] Camilleri J, Montesin FE, Curtis RV, Ford TR (2006). Characterization of Portland cement for use as a dental restorative material. Dent Mater.

[3] Islam I, Chng HK, Yap AU (2006). Comparison of the physical and mechanical properties of MTA and portland cement. J Endod.

[4] Menezes R, Bramante CM, Letra A, Carvalho VG, Garcia RB (2004). Histologic evaluation of pulpotomies in dog using two types of mineral trioxide aggregate and regular and white Portland cements as wound dressings. Oral Surg Oral Med Oral Pathol Oral Radiol Endod.

[5] Camilleri J, Montesin FE, Brady K, Sweeney R, Curtis RV, Ford TR (2005). The constitution of mineral trioxide aggregate. Dent Mater.

[6] Roberts HW, Toth JM, Berzins DW, Charlton DG (2008). Mineral trioxide aggregate material use in endodontic treatment: a review of the literature. Dent Mater.

[7] Dammaschke T, Gerth HU, Zu üchner H, Scha äfer E (2005). Chemical and physical surface and bulk material characterization of white ProRoot MTA and two Portland cements. Dent Mater.

[8] De-Deus G, Paciornik S, Mauricio MH (2006). Evaluation of the effect of EDTA, EDTAC and citric acid on the microhardness of root dentine. Int Endod J.

[9] Haapasalo M, Qian W, Portenier I, Waltimo T (2007). Effects of dentin on the antimicrobial properties of endodontic medicaments. J Endod.

[10] Zehnder M (2006). Root canal irrigants. J Endod.

[11] Smith JB, Loushine RJ, Weller RN, Rueggeberg FA, Whitford GM, Pashley DH (2007). Metrologic evaluation of the surface of white MTA after the use of two endodontic irrigants. J Endod.

[12] Yan P, Peng B, Fan B, Fan M, Bian Z (2006). The effects of sodium hypochlorite (5.25%), Chlorhexidine (2%), and Glyde File Prep on the bond strength of MTA-dentin. J Endod.

[13] Uyanik MO, Nagas E, Sahin C, Dagli F, Cehreli ZC (2009). Effects of different irrigation regimens on the sealing properties of repaired furcal perforations. Oral Surg Oral Med Oral Pathol Oral Radiol Endod.

[14] Khedmat S, Shokouhinejad N (2008). Comparison of the efficacy of three chelating agents in smear layer removal. J Endod.

[15] Yildirim T, Oruçoğlu H, Cobankara FK (2008). Long-term evaluation of the influence of smear layer on the apical sealing ability of MTA. J Endod.

[16] Estrela C, Estrada-Bernabé PF, de Almeida-Decurcio D, Almeida-Silva J, Rodrigues-Araújo-Estrela C, Poli-Figueiredo JA (2011). Microbial leakage of MTA, Portland cement, Sealapex and zinc oxide-eugenol as root-end filling materials. Med Oral Patol Oral Cir Bucal.

[17] Nekoofar MH, Namazikhah MS, Sheykhrezae MS, Mohammadi MM, Kazemi A, Aseeley Z (2009). pH of pus collected from periapical abscesses. Int EndodJ.

[18] Saghiri MA, Lotfi M, Saghiri AM, Vosoughhosseini S, Fatemi A, Shiezadeh V (2008). Effect of pH on sealing ability of white mineral trioxide aggregate as a root-end filling material. J Endod.

[19] Saghiri MA, Lotfi M, Saghiri AM, Vosoughhosseini S, Aeinehchi M, Ranjkesh B (2009). Scanning electron micrograph and surface hardness of mineral trioxide aggregate in the presence of alkaline pH. J Endod.

[20] Saghiri MA, Shokouhinejad N, Lotfi M, Aminsobhani M, Saghiri AM (2010). Push-out bond strength of mineral trioxide aggregate in the presence of alkaline pH. J Endod.

[21] Lotfi M, Vosoughhosseini S, Saghiri M, Zand V, Yavarí HR, Kimyai S (2011). Effect of alkaline ph on sealing ability of white mineral trioxide aggregate. Med Oral Patol Oral Cir Bucal.

[22] Namazikhah MS, Nekoofar MH, Sheykhrezae MS, Salariyeh S, Hayes SJ, Bryant ST (2008). The effect of pH on surface hardness and microstructure of mineral trioxide aggregate. Int Endod J.

[23] Bodanezi A, Carvalho N, Silva D, Bernardineli N, Bramante CM, Garcia RB (2008). Immediate and delayed solubility and mineral trioxide aggregate and Portland cement. J Appl Oral Sci.

